# Developmental status of human immunodeficiency virus-exposed uninfected premature infants compared with premature infants who are human immunodeficiency virus unexposed and uninfected

**DOI:** 10.4102/sajp.v76i1.1401

**Published:** 2020-06-18

**Authors:** Charne Cox, Joanne Potterton, Samantha Rosie

**Affiliations:** 1 Department of Physiotherapy, Faculty of Health Sciences, University of the Witwatersrand, Johannesburg, South Africa

**Keywords:** HIV exposed, premature, development, infants, neonatal complications, neonatal jaundice

## Abstract

**Background:**

There is growing concern about the developmental outcome of infants exposed to HIV in utero. HIV-infected women are at greater risk of premature delivery which poses a further developmental risk factor.

**Objectives:**

To determine whether there is a difference between the development of premature infants born at 28–37 weeks gestational age that are HIV exposed but uninfected (HEU) compared with HIV-unexposed uninfected infants (HUU).

**Method:**

A cross-sectional study was conducted in a Johannesburg state hospital. Thirty HEU and 30 HUU infants, aged between 16 days and six months, were assessed using the Bayley Scales of Infant and Toddler Development III.

**Results:**

The two groups were well matched for gestational age and birth weight; however, more HUU infants presented with neonatal complications. HUU infants had lower developmental scores than HEU infants in the language (*p* = 0.003) and motor (*p* = 0.037) subscales. Expressive language was more affected in the HUU infants (*p* = 0.001), and fine (*p* = 0.001) and gross motor (*p* = 0.03) were affected as well. HUU infants with neonatal complications such as meningitis (*p* = 0.02) and neonatal jaundice (NNJ) (*p* = 0.01) are more likely to present with language and motor delay.

**Conclusion:**

Meningitis and NNJ have more impact on infant development than in-utero HIV and ARV exposure.

**Clinical implications:**

It is important for all premature infants to be screened regularly in order to diagnose developmental delays early so as to ensure early intervention and improved quality of life.

## Introduction

South Africa is responsible for 70% of all new human immunodeficiency virus (HIV) infections globally (UNAIDS 2013). Human immunodeficiency virus-positive women who are not on antiretrovirals (ARVs) may transmit the infection to their babies during the *in utero* and intrapartum periods as well as postpartum through breastfeeding (Hilburn, Potterton & Stewart 2010). Human immunodeficiency virus mother-to-child transmission has been declining since 2008, when it was 8.5%. By 2016 it was reported to be 1.5% (Department of Health 2014, 2016). According to the World Health Organization (WHO), 73% of all pregnant women living with HIV in 2014 obtained medication to prevent transmission to their babies and 32% of children received HIV treatment.

An estimated 15 million babies are born prematurely every year (WHO, 2018). The incidence of preterm birth varies from one community to another and depends on a number of factors (Castell et al. 2013). These factors include low socio-economic status, poor maternal nutrition, maternal age, limited antenatal care, poor healthcare, heavy manual labour, maternal substance abuse, maternal hypertension, maternal diabetes, maternal stress and anxiety, twin pregnancies, trauma and infections (Castell et al. 2013; De Jongh et al. 2014; Irshad et al. 2012; Piscoya et al. 2012; Xu et al. 2015). Studies have shown that premature delivery is more frequent in HIV-positive women (Fiore et al. 2007; Powis et al. 2011; Traisathit et al. 2009). This could be because of severe immunosuppression from delayed antiretroviral therapy (ART) initiation, an increase risk for opportunistic infections as well as poor weight gain (Ezechi et al. 2013).

Premature infants are at an increased risk for growth failure, cerebral palsy, developmental delay and mental retardation (Saigal & Doyle 2008).

Many studies have found a correlation between HIV exposure and prematurity, low birth weights (LBW), intrauterine growth retardation, foetal death, neonatal mortality and respiratory complications (Darak et al. 2013; Nlend et al. 2016; Salihu et al. 2012). The developmental outcomes of HIV-exposed infants are still poorly researched and understood (Salihu et al. 2012).

Barret et al. (2003) found that infants exposed to ARVs during the perinatal period are at risk of poor neurological outcomes, such as cognitive delay, behavioural problems, motor abnormalities and convulsions, because of the continual mitochondrial dysfunction. The aim of our study was therefore to determine if there is a difference between the development of premature infants born at 28–37 weeks of gestational age that are HIV-exposed uninfected (HEU) compared to HIV-unexposed uninfected (HUU) infants at 16 days to 6 months of corrected age.

Very little research has been carried out on this topic in Southern Africa and not for infants aged 16 days to 6 months. If we can detect any developmental problem at a young age, we can begin early intervention at a younger age and potentially reduce the impact of HIV exposure on the child.

## Methodology

Our study was cross sectional. Participants were recruited from the neonatal follow-up clinic at Tambo Memorial Hospital (TMH) in South Africa. This clinic provides follow-up services for at-risk infants and includes those discharged from neonatal intensive care and those born prematurely.

A sample of convenience was used and the mothers of consecutive infants who met the inclusion criteria were invited to participate. Infants were included if they had a gestational age of 28–37 weeks and presented at the clinic between 16 days and 6 months of corrected age. HIV-exposed uninfected and HUU infants were eligible for inclusion. Infants were not eligible for inclusion if maternal HIV status was not known or if the infant presented with clinically obvious congenital conditions such as spina bifida.

A total of 60 infants (30 HEU and 30 HUU infants) met the inclusion criteria and their caregivers agreed to participate in the study. The sample size was calculated using the Bayley scales of Infant Development (BSID) III, 27 infants per group allowed for a power of 90% and a confidence interval of ± 4.63.

Infants were assessed using the BSID III in a quiet room. The BSID is considered to be the gold standard for infant developmental assessment globally. It has sound, well-established psychometric properties. The intra-rater reliability coefficients are excellent for the cognitive scale (Intraclass Correlation Coefficient (ICC) = 0.92–0.99) and good to excellent for the language (ICC = 0.88–0.98) and motor scale (ICC = 0.85–0.98). The inter-rater reliability coefficients are good to excellent for the cognitive (ICC = 0.87–0.97) and language scale (ICC = 0.76–0.95) and good for the motor scale (ICC = 0.77–0.89) (Yu, et al. 2013). Caregivers completed a demographic information form which took approximately 10 min and clinical history was obtained from the infant’s hospital file.

Descriptive statistics were conducted to condense the raw data with regard to information on the development of HUU and HEU infants. Tests used were means, standard deviation (SD) and confidence intervals (CIs). The level of significance was set at 0.05.

Inferential statistics were used to determine the differences between HUU and HEU infants. Test used was independent *t*-test as the groups are unrelated.

### Ethical consideration

Ethical clearance was obtained from the Human Research Ethics Committee (Medical) University of the Witwatersrand (clearance number: M160438). Caregivers of the infants provided informed consent to participate before any assessments were done.

## Results

The infants in the two groups were well matched for demographic information. The mean gestational age (SD) was 33.13 weeks (2.43) for the HEU group and 33.20 (2.57) weeks for the HUU group (*p* = 0.98). There were 43 (71.67%) infants in the late preterm category (32–27 weeks), 17 (28.33%) infants in the very preterm category (28–32 weeks) and no infants in the extremely preterm category (< 28 weeks). The mean corrected age in the HIV-exposed group at the time of assessment was 2.26 months and in the unexposed group it was 2.9 months (*p* = 0.09). The majority of infants were born with an LBW (67%) and there was no significant difference between the two groups for birth weight categories (*p* = 0.50) (see Table 1).

**TABLE 1:** Demographic information for human immunodeficiency virus -exposed uninfected and human immunodeficiency virus-unexposed uninfected groups.

Variable	HEU (*n* = 30)		HUU (*n* = 30)		*p*
	Number	%	Number	%	
**Birth weight**					
ELBW	2	6.7	3	3	0.50
VLBW	5	16.7	10	17	
LBW	20	66.7	40	67	
AVG	3	10	7	13	
**Gestational age**					
Mean	33.13	2.43	33.20	2.57	0.98
Median	33.5	-	33.00	-	

ELBW, extremely low birth weight; HEU, HIV-exposed uninfected; HUU, HIV-unexposed uninfected; VLBW, very low birth weight; LBW, low birth weight; AVG, average.

The clinical history of the two groups was similar for most factors assessed (see Table 2). A significant difference (*p* = 0.02) was seen in the number of infants who had a history of meningitis, with more HUU infants affected. Sixty-seven per cent of infants in the HUU group compared with 37% in the HEU group had a history of neonatal jaundice (NNJ) (*p* = 0.01).

**TABLE 2:** Complications for human immunodeficiency virus-exposed uninfected and human immunodeficiency virus-unexposed uninfected groups.

Variable	HEU (*n* = 30)		HUU (*n* = 30)		*p*
	Number	%	Number	%	
**Hydrocephalus**					
No	29	97	29	97	0.50
Yes	1	3	1	3	
**RDS/HMD**					
No	7	23	8	27	0.38
Yes	23	77	22	73	
**Mechanical Ventilation**					
No	18	60	23	77	0.08
Yes	12	40	7	23	
**Supplementary Oxygen**					
No	6	20	6	20	0.50
Yes	24	80	24	80	
**CLD**					
No	28	93	30	100	0.08
Yes	2	7	0	0	
**Pneumonia**					
No	26	87	29	97	0.08
Yes	4	13	1	3	
**NNJ**					
No	19	63	10	33	**0.01**
Yes	11	37	20	67	
**NNS**					
No	21	70	21	70	0.50
Yes	9	30	9	30	
**Meningitis**					
No	30	100	26	87	**0.02**
Yes	0	0	4	13	
**ICU admission**					
No	17	57	22	73	0.09
Yes	13	43	8	27	
**KMC**					
No	23	77	19	63	0.13
Yes	7	23	11	37	

RDS/HMD, respiratory distress syndrome/Hyaline membrane disease; CLD, chronic lung disease; NNJ, neonatal jaundice; NNS, neonatal sepsis; ICU, intensive care unit; KMC, Kangaroo mother care; HEU, HIV-exposed uninfected; HUU, HIV-unexposed uninfected.

Bold text describe the unexpected results from the standardised outcome measure used for these factors.

The composite scores of the BSID III were compared. There was no significant difference in the means of the cognitive composite scores between groups (*p* = 0.97). Language (*p* = 0.00) and motor (*p* = 0.04) composite scores showed a significant difference between the groups, with the HUU having lower developmental scores. All mean composite scores fell within 1 SD of the norm (Table 3).

**TABLE 3:** Developmental scores for human immunodeficiency virus-exposed uninfected and human immunodeficiency virus-unexposed uninfected premature infants.

Variable composite scores	HEU (*n* = 30)		HUU (*n* = 30)		*p*
	Mean	SD	Mean	SD	
Cognitive	98.20	13.68	98.30	20.14	0.97
Language	99.30	9.70	91.40	10.31	< 0.001*
Motor	104.70	12.95	96.70	15.77	0.04*

HEU, HIV-exposed uninfected; HUU, HIV-unexposed uninfected; SD, standard deviation.

* , Statistical significant difference.

When comparing expressive and receptive language scaled scores, the expressive language was more affected (*p* = 0.00) in the HUU infants. Scaled scores of both fine motor (*p* = 0.00) and gross motor (*p* = 0.03) were significantly lower in the HUU infants (Table 4).

**TABLE 4:** Scaled scores for human immunodeficiency virus-exposed uninfected and human immunodeficiency virus-unexposed uninfected premature infants.

Scaled scores	HEU (*n* = 30)		HUU (*n* = 30)		*p*
	Mean	SD	Mean	SD	
**Language**	19.9	3.40	17.0	3.56	< 0.001*
Expressive	10.8	1.95	8.4	2.19	< 0.001*
Receptive	9.0	1.71	8.6	2.16	0.40
**Motor**	22.3	2.30	18.8	5.21	< 0.001*
Fine	11.2	1.61	9.0	2.72	< 0.001*
Gross	11.1	1.31	9.8	2.83	0.03*

HEU, HIV-exposed uninfected; HUU, HIV-unexposed uninfected; SD, standard deviation.

* , Statistical significant difference.

Although there were no significant differences between the two groups, the HUU group was more delayed in the cognitive subscale (10%) and were at risk for delay in all subscales (6.7% cognitive, 20% language, 20% motor) when compared with the HEU group.

Because of the discrepancy in history of NNJ and meningitis between the two groups, these two factors warranted further investigation. The number of children in each group with meningitis was too low to be able to undertake further statistical analysis; however, the children with NNJ could be compared. Infants with a history of NNJ had significantly lower composite scores in the language (*p* = 0.00) and motor (*p* = 0.05) aspects of development.

Fifteen per cent of the HUU NNJ infants showed cognitive delay, with 10% being at risk. Five per cent of the HUU NNJ infants showed language delay with 25% being at risk. None of the HUU infants with a history of NNJ showed delay in motor development, but 25% were at risk (Table 5).

**TABLE 5:** Proportion of infants delayed or at risk for delay – Neonatal jaundice.

Subscale	Delayed (score < 70)		At risk (score 70–85)		TD (score > 85)		*p* (test difference in proportions in delayed between HEU and HUU)
	*n*	%	*n*	%	*n*	%	
**Cognitive**							0.09
HEU	0	0.0	1	9.1	10	90.9	
HUU	3	15.0	2	10.0	15	75.0	
**Language**							0.23
HEU	0	0.0	0	0.0	11	100.0	
HUU	1	5.0	5	25.0	14	70.0	
**Motor**							n/a
HEU	0	0.0	2	18.2	9	81.8	
HUU	0	0.0	5	25.0	15	75.0	

HEU, HIV-exposed uninfected; HUU, HIV-unexposed uninfected; n/a, not applicable; TD, Typical development.

## Discussion

Prematurity is a known risk factor for developmental delay (Guimaraes et al. 2011; Nepomnyaschy et al. 2012) and it affected both groups equally. Research has shown that pregnancy outcomes such as premature births (PTB) and LBW are associated with Protease inhibitor-based ART as well as the timing of the initiation of the ART (Darak et al. 2013; Mesfin, Kibret & Taye 2016; Powis et al. 2011). A study conducted by Mesfin et al. (2016) showed that there is a 32% increased risk of PTB when a mother receives these Protease Inhibitor (PI)-based ART regimes, especially if it is given within the first trimester (< 13 weeks) and the third trimester. This is important as all the mothers in our study were receiving ART (Darak et al. 2013; Powis et al. 2011). Another study conducted by Darak et al. (2013) showed that 45% of women in their study had adverse pregnancy outcomes whereby there were 17% PTB and 27% LBW.

Most infants were born with an LBW (1500 g – 2499 g). Birth weight has been shown to have a negative impact on development (Ballot et al. 2012; Chaudhury et al. 2017; Oudgenoeg-Paz et al. 2017). It was found that children born with an LBW are at risk for poor quality of movement and postural control. It is this affected early motor development that may affect cognitive development at a later stage (Oudgenoeg-Paz et al. 2017). Low birth weight is a risk factor that affected both groups in our study with regard to motor and cognitive development.

Meningitis and neonatal jaundice are independent well-documented causes of neurological impairment in neonates (Heath & Okike 2010; Ku, Boggess & Cohen-Wolkowiez 2015; Rosie, Potterton & Pilusa 2016). In this particular group of infants, the HUU group had a higher prevalence of NNJ. A study conducted at Baragwanath Hospital in Soweto, South Africa, which investigated the neurodevelopmental outcome of 40 Neonatal Intensive Care Unit (NICU) survivors (mean gestational age 34 weeks), found that NNJ was a risk factor for abnormal language and motor development (Rosie, Potterton & Pilusa 2016). This is similar to the findings in our study.

Recent studies have shown that HEU infants are at risk of opportunistic infections, such as pneumonia, tuberculosis and diarrhoea, all of which can have a major impact on the health and development of an infant (Evans, Jones & Prendergast 2016; Rajan et al. 2017). This is in contrast to our study as complications such as pneumonia showed no significant differences between the two groups.

The developmental scores showed that the majority of the HEU infants did well in all aspects of development when compared with the HUU group. The mean developmental scores for the HEU infants were all within 1 SD of the norm and therefore showed typical development in this group of infants.

Much debate on the impact of intra-uterine exposure to HIV as well as ARVs still exists. Most of the studies that have been conducted have included infants born at term with little consideration of premature infants. A study conducted in India by Rajan et al. (2017) reported on the developmental outcome of 41 HEU infants aged 6–18 months, of whom 40 had typical development. This study concluded that the ARV drug exposure *in utero* had no effect on the development of these infants.

A South African study conducted in Cape Town in 2017 assessed the development of HEU infants at 12 months of age using the BSID III. The authors found there is a minor difference in neurodevelopmental outcome when compared with HUU infants (Springer et al. 2018). In contrast to our study, Springer et al. (2018) found that a higher proportion of HEU infants had a poorer developmental outcome with regard to language development. This study did not take prematurity into consideration and looked at other risk factors, such as socio-economic characteristics, socio-demographic characteristics and anthropometric characteristics.

When looking at a study with older HEU children (24 months) in Botswana, there is still no negative neurodevelopmental outcome when assessed with the BSID III. They assessed 313 HEU infants and concluded that there was no negative neurodevelopmental impact on children when they are exposed to the HIV virus *in utero* or the ARV drugs (Chaudhury et al. 2017).

In our study, the HUU infants had lower developmental scores in expressive language as well as fine and gross motor subscales of the BSID III. This group had more neonatal complications such as meningitis and NNJ, and this seems to have had more of an effect on the infant’s central nervous system (CNS) than HIV exposure.

Human immunodeficiency virus-exposed uninfected infants are likely to be born prematurely because of the ARVs during pregnancy in order for Prevention of Mother to Child Transmission (PMTCT) (Nlend et al. 2016); however, HUU infants are born prematurely when other complications or lifestyles arise (Saltani et al. 2017; Waetcher 2014).

Meningitis is a bacterial infection of the CNS and has the highest incidence in the neonatal period. The risk factors include prematurity, LBW, chorioamnionitis, prolonged rupture of membrane, premature rupture of membrane, low socio-economic status, need for resuscitation, prolonged hospitalisation and male infants (Heath & Okike 2010; Ku, Boggess & Cohen-Wolkowiez 2015). The more premature the infant, the worse the prognosis is and the more likely the infant will present with long-term neurodevelopmental complications (Heath & Okike 2010; Ku, Boggess & Cohen-Wolkowiez 2015; Lin et al. 2012).

High levels of circulating bilirubin in neonates can have severe and serious consequences as the bilirubin can cross the blood–brain barrier and attach to the developing brain tissue. The most vulnerable areas of the infant’s brain are those that control hearing and motor function (Casey 2013; Pearsall & Morrow 2016).

Studies have shown that NNJ has an effect on the development of infants. Rosie et al. (2016) found that 37.5% of their 40 neonatal ICU survivors (mean gestational age 34 weeks) were treated for NNJ. These infants were found to be at risk for language and motor developmental delays. This was confirmed in our study as 25% of HUU infants with NNJ were at risk for language and motor delays, with only 10% being at risk for cognitive delay.

It would have been beneficial to follow up the infants at a later stage to determine whether their development would improve at a later age as the infants were very young. The small sample size and many possible confounding factors may have influenced the results of our study.

Our results suggest that HIV and ARV exposure *in utero* seem to have little effect on the development of an infant. Birth complications such as meningitis and NNJ have a much bigger negative impact on neonatal development. All premature infants, whether HEU or HUU, should have developmental follow-up post-discharge to ensure early intervention if the need arises.

Antiretrovirals in pregnancy are critical in preventing mother-to-child transmission of HIV and ensuring a healthier infant with less neurodevelopmental complications. Larger long-term follow-up studies are needed to monitor the developmental trajectories of infants exposed to HIV and ARVs *in utero*.
